# COVID-19-Related ARDS: Key Mechanistic Features and Treatments

**DOI:** 10.3390/jcm11164896

**Published:** 2022-08-20

**Authors:** John Selickman, Charikleia S. Vrettou, Spyros D. Mentzelopoulos, John J. Marini

**Affiliations:** 1 Department of Pulmonary and Critical Care Medicine, University of Minnesota School of Medicine, Minneapolis, MN 55455, USA; 2 First Department of Intensive Care Medicine, National and Kapodistrian University of Athens Medical School, Evaggelismos General Hospital, 10676 Athens, Greece; 3 Department of Pulmonary and Critical Care Medicine, Regions Hospital, St. Paul, MN 55101, USA

**Keywords:** COVID-19, acute respiratory distress syndrome, mechanical ventilation, SARS-CoV-2

## Abstract

Acute respiratory distress syndrome (ARDS) is a heterogeneous syndrome historically characterized by the presence of severe hypoxemia, high-permeability pulmonary edema manifesting as diffuse alveolar infiltrate on chest radiograph, and reduced compliance of the integrated respiratory system as a result of widespread compressive atelectasis and fluid-filled alveoli. Coronavirus disease 19 (COVID-19)-associated ARDS (C-ARDS) is a novel etiology caused by severe acute respiratory syndrome coronavirus 2 (SARS-CoV-2) that may present with distinct clinical features as a result of the viral pathobiology unique to SARS-CoV-2. In particular, severe injury to the pulmonary vascular endothelium, accompanied by the presence of diffuse microthrombi in the pulmonary microcirculation, can lead to a clinical presentation in which the severity of impaired gas exchange becomes uncoupled from lung capacity and respiratory mechanics. The purpose of this review is to highlight the key mechanistic features of C-ARDS and to discuss the implications these features have on its treatment. In some patients with C-ARDS, rigid adherence to guidelines derived from clinical trials in the pre-COVID era may not be appropriate.

## 1. Introduction

Acute respiratory distress syndrome (ARDS), as initially described, defined patients with similar clinical and pathologic findings: refractory hypoxemia; diffuse alveolar infiltrates on chest X-ray; severely reduced lung compliance; and, in those who did not survive, heavy lungs at autopsy, characterized by diffuse alveolar injury with hyaline membranes [1]. As its manifestations appeared superficially similar regardless of etiology, it was reasoned that treatment should be essentially the same, and this quickly became a universally accepted approach [2].

Randomized trials of therapy for such a pathologically and mechanically defined entity, however, might require up to one thousand patients to demonstrate survival benefit. Consequently, the need to establish definitions broad enough to permit sufficient enrollment with smaller numbers became evident [3]. Definitional simplification that excluded such hallmark features of ARDS as low respiratory compliance succeeded in facilitating enrollment for clinical studies, of course, but this came at the expense of specificity. In a recent study, for example, 14% of patients meeting the definition for ARDS had *no identifiable pulmonary lesions* at post mortem examination [4]. As a result, randomized trials incorporating these simplified definitions have included patients with an extraordinary range of respiratory mechanics and severity of illness. Yet, conducting such trials has led to the impression among many clinicians that “ARDS” represents a distinct disease-like entity [5].

Whether all patients with ARDS should be treated similarly and without discrimination regarding etiology is not a new question but rather one with renewed immediacy. The COVID-19 pandemic, caused by severe acute respiratory syndrome coronavirus 2 (SARS-CoV-2), has overwhelmed intensive care units with cases of respiratory failure meeting the broadened diagnostic criteria for ARDS [6]. While the number of patients requiring invasive mechanical ventilation for ARDS secondary to COVID-19 (C-ARDS) has declined over time [7], mortality in this population remains high [8]. Many have argued that C-ARDS should be managed no differently than ARDS of any other etiology, ignoring that the viral pathogenesis of SARS-CoV-2 may lead to a distinct form of ARDS that diverges from “typical” ARDS. For C-ARDS patients requiring mechanical ventilation, current guidelines, derived from studies of ARDS primarily caused by bacterial pneumonia and septic abdominal disease [9], may not be universally appropriate.

The purpose of this review is to describe how the physiology of C-ARDS, generated by the unique viral pathobiology of SARS-CoV-2, may differ from non-COVID ARDS, emphasizing the implications of that difference for both pharmacotherapy and mechanical ventilation. We underline that rigid adherence to all pre-COVID ventilatory guidelines may be ill-advised. Finally, we discuss management of refractory C-ARDS and the role of extracorporeal life support.

## 2. “Typical” ARDS

ARDS is currently defined by the Berlin Definition (Table 1) [6] and is characterized by high-permeability pulmonary edema and widespread compressive atelectasis. In response to injury, immune cells trigger an inflammatory response that leads to disruption of the alveolar–capillary barrier [10]. Accumulation of protein-rich fluid in alveolar and interstitial spaces inhibits pulmonary surfactant [11] which, along with increased hydrostatic pressures from extravascular lung water, results in collapse of underlying lung units. Physiologically, this manifests as (1) severely impaired gas exchange, with refractory hypoxemia and hypercarbia secondary to intrapulmonary shunt and reduced functioning surface for gas exchange [12,13,14]; and (2) severely reduced lung compliance. Histologically, this initial phase manifests as “diffuse alveolar damage,” a constellation of findings involving damage to the alveolar lining and endothelium, the presence of hyaline membranes, interstitial and alveolar edema, and inflammatory infiltrate [15].

Studies using quantitative computerized tomography (CT) have demonstrated that not only is the ARDS lung heterogeneous, with normally aerated units co-existing alongside non-aerated ones [16], but that the location of non-aerated units is strongly influenced by gravity, owing to the compressive forces of overlying edematous lung tissue. For that reason, radiographic densities appear to migrate from the paravertebral region when supine to the parasternal region when prone [17]. These studies have further shown that, in ARDS, compliance of the integrated respiratory system is determined primarily by the number of aerated lung units [18]; in other words, low compliance in ARDS is due in large part to the lungs being “small” not “stiff” [19]. Collectively, these findings gave rise to the concept of the “baby lung”, a construct drawing similarity between the volume of aerated tissue in the low-capacity lung of ARDS and the volume of aerated tissue in the lung of a healthy child [20]. As total chest dimensions remain unaltered by ARDS, tissue density inversely parallels the capacity of the baby lung.

This concept has important implications. First, the severity of gas exchange impairment is intrinsically linked to the quantity of non-aerated tissue, with shunt fraction and physiologic dead space increasing, and PaO_2_ decreasing, as the percentage of non-aerated lung rises [21]. Therefore, in typical ARDS, oxygenation and compliance are expected to deteriorate in direct proportion to one another. Additionally, the loss of ventilatory capacity means that the entire workload of ventilation is concentrated in an overtaxed baby lung, increasing the risk for progressive injury and further loss of functional lung units [22]. Protective strategies for ventilation have therefore been directed towards expanding the size of the baby lung through alveolar recruitment with the intent of distributing workload among a greater number of functional lung units, while avoiding exposure to (and unnecessary repetition of) tidal cycles that excessively strain vulnerable structural microelements.

While imperfect, as there is significant heterogeneity within the ARDS population, we use the term “typical” to collectively refer to ARDS described in the pre-COVID ARDS literature, which predominantly focused on patients with bacterial pneumonia and intra-abdominal disease.

## 3. Viral Pathogenesis of SARS-CoV-2

Appreciation for the viral pathogenesis unique to SARS-CoV-2 underpins a solid understanding of physiologic disparities between C-ARDS and non-COVID ARDS. SARS-CoV-2 expresses multiple structural proteins on its viral envelope, including the spike protein, a glycoprotein that mediates binding to host cells [23]. Cellular tropism is determined not only by the expression of angiotensin converting enzyme 2 (ACE2) receptors on the surface of host cells [24], which the spike protein binds to directly, but also the presence of transmembrane serine protease (TMPRSS2), which cleaves spike protein and facilitates viral uptake [25]. Following the release of the viral ribonucleoprotein into the cytoplasm, viral replicases use endoplasmic reticulum membranes to form double membrane vesicles for ″protected″ viral RNA transcription (termed replication factories) [26,27].

ACE2 receptors are expressed widely throughout the body, but their concentration is especially high in the pulmonary vascular endothelium and respiratory tract. As a result, the cells first targeted by SARS-CoV-2 following inhalation are those located in the nasopharynx and upper airway (e.g., multiciliated cells or sustentacular cells of the olfactory mucosa) [28,29]. When host immunity fails to clear SARS-CoV-2 infection, it spreads to the lower respiratory tract, either by aspiration of viral particles from the oropharynx or gradual progression throughout the tracheobronchial tree; in some cases, it may bypass the upper respiratory tract altogether [30]. Upon reaching the alveoli, SARS-CoV-2 primarily affects alveolar type 2 (AT2) cells which, in health, are tasked with both production of pulmonary surfactant and regeneration of AT1 cells (which constitute the majority of the alveolar epithelium) [31].

Following infection, host cells initially attempt to control viral spread through innate immunity. Cytoplasmic pattern recognition proteins detect RNA fragments of SARS-CoV-2, triggering the release of interferons, pro-inflammatory cytokines and leukocyte recruitment [32]; additional cytokine release occurs when damage-associated molecular patterns in host cells are released in response to injury [33]. If the innate immune response is dysfunctional, infection will spread, increasing the risk for severe COVID-19; alternatively, if the adaptive B and T cell responses to innate cytokine and chemokine release are absent, uncontrolled inflammation may ensue [34].

**Figure 1:**
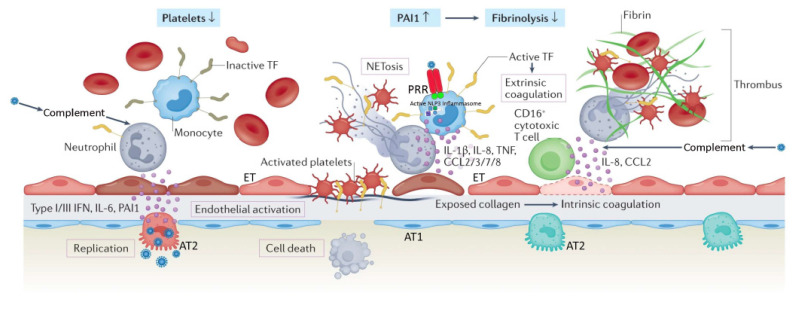
Severe coronavirus disease 19 is characterized by immune cell-mediated hypercoagulability and hypofibrinolysis. Hypoxia, cytokines, chemokines, damage-associated molecular patterns, and direct infection by the virus contribute to alveolar and endothelial cell death, and disruption of the alveolar–capillary barrier. Exposed extracellular matrix can trigger both the extrinsic coagulation (via tissue factor (TF)) and the intrinsic coagulation (via collagen/RNA). Recruited monocytes (with virus-activated NLP3 inflammasomes) and neutrophils amplify the inflammatory response, as well as the activation of coagulation by expressing active tissue factor (TF) and releasing neutrophil extracellular traps (NETs), respectively. Complement activation by the virus promotes active TF expression by neutrophils, and differentiation of cytotoxic CD-16^+^ T cells. NETs recruit platelets, which are subsequently activated by NET histones and the C3a and C5a complement fragments; this results in platelet release of cytokines. Activated platelets secrete coagulation-sustaining factors. The immunothrombotic process leads to diffuse small-vessel thromboses and thrombocytopenia. Concurrently, increased expression of plasminogen activator inhibitor (PA1) attenuates fibrinolysis. *AT1*, alveolar type 1 cell; *AT2*, alveolar type 2 cell; *ET*, endothelial cell; *PRR*, pattern recognition receptor; *IL*, interleukin; *CCL*, CC chemokine ligand; *IFN*, interferon. Reproduced in part with permission from [26]; copyright (2022) by Springer Nature.

Alveolar cell injury or death causes disruption of the alveolar epithelium, thereby setting off an imbalance between coagulation activation and fibrinolysis [26,35]. Fibrin-rich alveolar exudates form hyaline membranes, which prevent further fluid accumulation into the injured alveoli but also hinder the alveolar–capillary oxygen transport [26,36]. Diffuse alveolar damage is followed by small-vessel endothelial activation and injury secondary to hypoxia, cytokines, chemokines, damage-associated molecular patterns, and direct infection by the virus [26,37,38]. Diffuse endotheliitis with inflammatory cell infiltrates may induce widespread endothelial cell apoptosis, pyroptosis, and microcirculatory dysfunction contributing to C-ARDS and also promoting extrapulmonary organ/system failure [26,37]. Release of the endothelial tissue factor can activate the extrinsic coagulation pathway [39]. Extracellular RNA, DNA, and exposed collagen can also activate factor XII and the intrinsic coagulation pathway [40]. Concurrently, platelets seal off the area of endothelial damage to prevent vascular leakage and secrete coagulation-sustaining factors [41] (Figure 1).

In the context of COVID-19 immunothrombosis (Figure 1), recognition of SARS-CoV-2 through pattern recognition receptors of monocytes results in the release of activated tissue factor at sites of virus localization [26,42]. SARS-CoV-2 stimulates the NLPR3 inflammasome, with consequent production of interleukin (IL) 1 beta (IL-1-β) and IL-18 [42,43]. Concurrently, there is increased release of IL-6 from the alveolar epithelium, which in turn stimulates the production of clotting factors in the liver and tissue factor in the endothelium [26]. Complement activation by SARS-CoV-2 results in (1) upregulated expression of tissue factor by neutrophils [26,44]; and (2) differentiation of a CD-16 expressing T cell subpopulation, promoting immune complex-induced degranulation, microvascular endothelial cell injury, and release of IL-8 and chemokines [26,45]. Activated neutrophils release neutrophil extracellular traps (NETs), which directly activate factor XII and bind von Willebrand factor to promote recruitment of platelets [42]. NET histones, and complement fragments C3a and C5a, activate platelets, while neutrophil elastase and myeloperoxidase inactivate anticoagulants such as tissue factor pathway inhibitor [42]. NET-associated platelets activate the intrinsic coagulation pathway and release large amounts of pro-inflammatory cytokines [42]. Immune-activated platelets (through pattern recognition receptors) also propagate the innate immune response and immunothrombosis by releasing platelet factor 4 and high-mobility group box 1 protein, as well as platelet-derived extracellular vesicles [46]. Immunothrombotic mechanisms are further enhanced by hypofibrinolyis secondary to increased expression of plasminogen activator inhibitor [26,47] (Figure 1).

Systemic hyperinflammation is the *sine qua non* of C-ARDS and may be especially prominent in subsets of patients with (1) risk factors such as age, obesity, cardio-respiratory comorbidity, diabetes, and immunosuppression [26,48,49]; (2) genetic predisposition (e.g., variants at chemokine receptor genes or genes involved in interferon induction and amplification) [26,50,51,52,53,54]; and (3) autoantibodies against type I interferons [55,56]. Besides C-ARDS, severe and potentially lethal COVID-19 may also have extrapulmonary manifestations including gastrointestinal symptoms, acute cardiac, renal, and liver injury, rhabdomyolysis, coagulopathy, cardiac arrhythmias, and circulatory failure [26,57]. Lastly, while some studies have demonstrated systemic inflammation in C-ARDS to be less robust than non-COVID ARDS [58,59], pro-inflammatory responses are tightly linked to injury of the pulmonary vascular endothelium and immunothrombosis, both of which are distinct pathophysiologic features of C-ARDS in terms of their severity and ubiquity [30].

## 4. Pharmacologic Interventions

Despite hypercoagulability, full therapeutic anticoagulation did not prove superior to prophylactic anticoagulation in an international randomized controlled trial (RCT) of severe COVID-19 [60]. In contrast, treatments focusing on the inflammatory component of COVID-19 thrombosis have been repeatedly associated with improved patient outcomes. Indeed, “general inhibition” of inflammatory processes with dexamethasone or hydrocortisone resulted in a 30–36% reduction in the odds for in-hospital death of critically ill COVID-19 patients [61,62].

Despite initially discouraging findings [63], a meta-analysis of 19 RCTs reported a 17% reduction in the odds for in-hospital mortality with the IL-6 antagonist tocilizumab compared to usual care or placebo [64]. When compared to usual care, the addition of baracitinib, a janus kinase inhibitor, also resulted in shorter recovery time [65], reduced mortality [66], and lower frequency of adverse events [67]; furthermore, in a meta-analysis of four RCTs, baracitinib was associated with a 31% reduction in the odds for in-hospital death [68]. Guided by soluble urokinase plasminogen receptor plasma levels, treatment with IL-1 alpha and IL-1 beta antagonists also resulted in a 64% reduction in clinically worsened status at day 28, less organ dysfunction at day 7, and lower in-hospital mortality [69].

Collectively, these results highlight not only the clinical relevance of inhibiting key inflammatory processes that contribute to widespread endothelial dysfunction, diffuse small-vessel thromboses, and multiorgan failure, but also a broader theme—the treatment of C-ARDS, whether it be pharmacologic or otherwise, diverges from the treatment of non-COVID ARDS.

## 5. Blood Purification Interventions

In septic shock, extracorporeal cytokine removal with Cytosorb has been associated with lower IL-6 levels [70], reduced norepinephrine requirements [71], and lower observed vs. expected, 28-day, all-cause mortality [70,72]. These data, along with the recently proven efficacy of immunomodulating agents and the potentially beneficial effects reported by COVID-19 case series [73,74], supported the hypothesis that cytokine adsorption might improve severe COVID-19 outcomes [75,76]. However, in a small RCT of severe C-ARDS requiring extracorporeal membrane oxygenation (ECMO), patients treated with Cytosorb for 72 h had similar IL-6 concentrations and higher 30-day mortality compared to control [75]. Furthermore, in a second RCT of COVID-19 patients with vasoplegic shock, Cytosorb treatment for 3–7 days did not expedite shock reversal, and had no significant effect on markers of inflammation, vasopressor requirements, and 90-day mortality [76]. Notably, these findings are consistent with the results of two prior, small RCTs in septic or cardiac surgery patients [77,78]. Collectively, Cytosorb RCTs have failed to demonstrate any clinically meaningful difference between intervention and control groups [75]. Any previously observed cytokine lowering and hemodynamic stabilization are likely attributable to the natural course of the disease and adjunctive therapy rather than non-specific cytokine adsorption [75]. Alternative approaches to extracorporeal blood purification such as heparin-functionalized adsorbents are currently under evaluation with respect to their efficacy in depleting pathogens and mediators of immunothrombosis [46].

## 6. Distinct Pathologic Features of C-ARDS

Substantial clinical and biologic heterogeneity exists within the ARDS population [79]. Subphenotypes with distinct clinical features and responses to therapy have been identified with respect to the initial site of injury (pulmonary or extrapulmonary) [80] and biologic markers of inflammation (hypo- or hyperinflammatory) [81]. It should thus come as little surprise that properties unique to the SARS-CoV-2 virus itself might result in a form of ARDS with distinctive pathophysiology, or that even amongst patients with ARDS of a single etiology (e.g., C-ARDS), there might be a significant diversity of findings and responses to treatment (Table 2).

Reports comparing the pathologic features of C-ARDS to other forms of viral or non-viral ARDS are fraught with conflicting results, as accounting for the stage of disease and evolution of practice patterns over time is challenging. One theme that has consistently emerged, however, is the near-universal presence of pulmonary vascular abnormalities in patients with C-ARDS [82].

Though often present, pulmonary vascular lesions are not a dominant histopathologic feature of usual ARDS and are seldom widespread in post mortem lung specimens [15,83]. In patients with C-ARDS, however, they not only occur commonly [84,85] but are extensive, occupying greater than 25% of the lung parenchyma in over half of the patients examined at autopsy in one study [86]. While microvascular thrombi may be a shared histologic finding among all patients with ARDS caused by pulmonary viruses, including influenza A and SARS-CoV-1 [82], the extent of microthrombosis appears to be far greater in patients with C-ARDS [38]. This prevalence tends to uncouple gas exchange from mechanical properties, calling into question the specifics of ventilation management guidelines developed from clinical trials in the non-C-ARDS setting. Furthermore, the thrombotic burden is not confined to the microcirculation; the incidence of large-vessel pulmonary emboli is higher in patients with C-ARDS than in those of ARDS secondary to other viral and non-viral etiologies [87,88]. Other pulmonary vascular derangements observed at autopsy include severe endothelial injury [37,38] and the presence of dilated/engorged capillaries [89].

Studies incorporating dual-energy computerized tomographic angiography (CTA), digital subtraction CTA, and high-resolution CT have further extended these findings. Pulmonary vascular abnormalities on CT, most notably vessel enlargement, are common in patients with COVID-19 and may even be present prior to the development of C-ARDS [90]. Enlarged vessels suggestive of vasodilatation can be frequently observed within an area of ground glass or consolidation [91], contrary to the expected physiologic response to regional hypoxia (i.e., vasoconstriction). Perfusion imaging confirms that a considerable fraction of opacified lung parenchyma demonstrates increased uptake (indicating blood flow) in spite of diminished or even absent ventilation [92]. Perfusion abnormalities, on the other hand, are detected in areas of *normal lung density* [90], with one study of mechanically ventilated C-ARDS patients reporting that perfusion defects were not only present in every patient studied, but that the median extent of vascular abnormality approached 50% [93].

## 7. Respiratory Mechanics and Gas Exchange in C-ARDS

Early in the pandemic, Gattinoni and colleagues reported novel findings in their first 16 patients with C-ARDS; these patients had a relatively *high* tidal compliance (averaging 50.2 mL/cm H_2_O) associated with significantly elevated shunt fraction (0.50) [94]; furthermore, in the eight patients they evaluated using quantitative CT, the ratio of shunt fraction to gasless tissue was markedly higher (roughly 2.5 times) than those observed in usual ARDS [95], consistent with hyperperfusion of gasless tissue.

Chiumello and colleagues performed similar quantitative CT analysis in 32 consecutive C-ARDS patients receiving mechanical ventilation and compared gas exchange, respiratory mechanics, and CT variables to those of two historical cohorts of usual ARDS: one matched 1:1 for PaO_2_/FiO_2_ (P/F) and one matched 1:1 for compliance [96]. Compared to the C-ARDS cohort, the historical ARDS cohort matched for P/F had significantly lower compliance values (39.9 versus 49.4 mL/cmH_2_O) and gas volumes on CT (930 mL versus 1670 mL). The historical ARDS cohort matched for compliance, on the other hand, had a higher P/F when compared to the C-ARDS cohort (160 versus 106.5 mmHg).

These findings are well explained by the pulmonary vasculopathy and diffuse, inflammation-triggered microthrombosis observed in COVID-related lung disease. In typical ARDS, airspace flooding, collapse, and consolidation tend to parallel the severity of oxygenation impairment and fall in compliance. C-ARDS challenges this conceptual framework; specifically, lung compliance may be well preserved in the early and mild stages of C-ARDS (at least in a major fraction of these patients), with severe hypoxemia not occurring primarily as a result of airspace filling and lung unit drop-out, but as the consequence of increased perfusion to non-ventilated lung units [89,97,98,99,100]. Over time, however, progression of C-ARDS fundamentally alters the lung’s mechanical properties. In late phase ARDS, regardless of the cause, lung capacity becomes severely reduced and is characterized by high dead space, limited recruitability, and low compliance [101].

As might be expected from the loosely defined and oxygenation-based criteria for ARDS and the evolving nature of COVID-related lung injury, there is wide overlap between the mechanics of C-ARDS and usual ARDS; indeed, several studies evaluating their comparative mechanical properties did not identify distinctive mean differences between cohorts [102,103], which may in part be a function of the stage of illness in which such observations were made [104,105].

## 8. Mechanical Ventilation in C-ARDS

The goals of invasive mechanical ventilation in C-ARDS are to relieve excessive work of breathing, improve gas exchange, and avoid aggravation of existing lung injury. Repeated exposure to tidal cycles that cause excessive, fracturing strain of structural microelements is believed to be the proximate mechanical stimulus for ventilator-induced lung injury (VILI) [106]; in recent years, a better understanding of the biophysical causes of VILI has shifted our traditional focus from the inflation pattern of a single tidal cycle toward avoiding exposure to damaging levels of tidal energy and power [107]. At the bedside, however, the focus remains on attempting to restrain tidal plateau and driving pressures below defined numerical thresholds. Unfortunately, this well-intentioned objective is often pursued through application of inflexible ventilatory targets and without consideration of the stage of disease.

In many patients with C-ARDS, ventilator strategies shown to be beneficial in clinical trials of unselected patients with ARDS will be appropriate; for others, however, they may not apply. The body of C-ARDS literature has expanded at a remarkable pace throughout the pandemic, providing guidance in certain areas regarding optimal ventilator management. Knowledge gained through physiologic studies preceding the C-ARDS era must be applied judiciously in order to provide individualized care for patients with ARDS of any etiology—including those with COVID-19.

### 8.1. Tidal Volume in C-ARDS

Twenty years ago, the ARMA trial [9] demonstrated a 9% absolute reduction in mortality among mechanically ventilated ARDS patients randomized to an initial tidal volume of 6 mL/kg predicted body weight, forming the basis for what has become a standard of care codified in most ARDS guidelines [108,109]. While large tidal volumes that lead to excessive strain are undoubtedly misguided in any acutely injured lung [110], several points are worth noting with respect to tidal volume selection in C-ARDS:

(1) Data from the ARMA trial, derived primarily from patients with ARDS secondary to bacterial pneumonia and sepsis, may not be wholly translatable to patients with ARDS secondary to novel forms of viral pneumonia with unique pathologic features, such as C-ARDS.
(2) Even in the ARMA trial, tidal volumes could be liberalized if necessary to facilitate patient comfort and adequate ventilation.
(3) In three large randomized trials that preceded the ARMA trial, no differences were found between patients treated with means of 7.2 mL/kg versus 10.6 mL/kg predicted body weight [111]; 7.2 mL/kg versus 10.4 mL/kg dry body weight [112]; and 7.3 mL/kg versus 10.2 mL/kg predicted body weight [113].

In the subpopulation of C-ARDS patients with less alveolar injury and relatively preserved compliance, larger tidal volumes of 7–8 mL/kg predicted body weight may result in tolerable strain and energy input without the risk of VILI [107]. In such patients, enforcing low tidal volumes can unnecessarily increase dead space [114], lead to reabsorption atelectasis from hypoventilation, and necessitate additional sedation to facilitate breathing comfort. However, as the severity of disease progresses and compliance declines, lower tidal volumes may be required to prevent the generation of strain that exceeds critical thresholds of injury.

### 8.2. Application of PEEP in C-ARDS

Since the severity of gas exchange impairment and loss of compliance in the baby lung of ARDS reflect the reduced number of lung units available to accept ventilation, it is logical that interventions leading to an increase in the number of functional lung units should improve hypoxemia, reduce dead space, and increase compliance. Positive end-expiratory pressure (PEEP) is applied with the intent of achieving these goals by preventing collapse of unstable alveoli and thereby stabilizing “recruitment.” Expanding the ventilatory capacity in this manner additionally serves to distribute energy across a greater number of lung units, perhaps decreasing the quantity of damaging tidal energy transferred to the parenchymal matrix and reducing the risk of VILI [19].

Employing PEEP for the purposes of alveolar recruitment, however, hinges on the assumptions that compromised gas exchange is due primarily to loss of otherwise functional lung units and that these collapsed, or fluid-filled, units will regain function in response to the application of end-expiratory pressure. In C-ARDS, these assumptions may not hold true, and if they do, may be strongly dependent on the timing of the intervention [115].

Within the baby lung, the regional effects of PEEP are highly variable, as both recruitment and overdistension occur simultaneously as the lung expands. The net benefit of PEEP depends on whether recruitment of functional lung units outweighs overdistension within those that were already functional. When overdistension prevails, gas exchange is adversely affected as blood flow is directed away from overdistended lung units that previously participated in gas exchange, resulting in increased dead space and encouraging hypercarbia. The effects of net overdistension on oxygenation, on the other hand, are variable. Oxygenation may initially improve in response to increased PEEP despite net overdistension, especially if decreased cardiac output leads to reduction in blood flow through areas of intrapulmonary shunt, making the P/F ratio a poor surrogate for recruitment [116,117].

When PEEP results in significant net recruitment, respiratory compliance (a correlate of baby lung size) will improve. However, when PEEP results in significant net overdistension, compliance will fall as open lung units are shifted past the upper inflection point of their pressure–volume curve. Under these conditions, the increased energy input associated with higher PEEP serves only to increase the risk of VILI and hemodynamic perturbations [118].

In recent decades, lung-protective strategies have focused on not only the use of low tidal volumes for ventilation, but also the application of higher PEEP [108]. “PEEP tables,” in which PEEP is increased in a stepwise fashion with respect to the inspired oxygen requirement, assume that impaired oxygenation is secondary to the loss of functional lung units. Based on their use in clinical trials, such tables are commonly used by clinicians managing ARDS to select PEEP [119]. In many centers, this practice resulted in the early use of PEEP levels exceeding 14 cmH_2_O for C-ARDS [120]. In C-ARDS, however, impaired oxygen exchange is often strongly influenced by vascular dysfunction—not loss of functional lung units—in which case high levels of PEEP are not beneficial. In one study of mechanically ventilated patients with C-ARDS, partitioned respiratory mechanics were measured at low and high levels of PEEP [121]. Compared to 5 cmH_2_O, a PEEP of 15 cmH_2_O resulted in reduced lung compliance, increased lung strain, and an increased ventilatory ratio (i.e., a surrogate of physiological dead space defined as the quotient of measured over predicted product of minute ventilation and PaCO_2_ [122]). Had PEEP in that study [121] been set in accordance with the P/F table used in a recent clinical trial [123], it would have been 18 cmH_2_O.

While response to PEEP varies significantly among individual patients with C-ARDS [100], functional recruitment appears to be diminished relative to usual ARDS [96] and likely is influenced by the stage of disease and timing of observation [124]. Studies incorporating quantitative CT have either demonstrated minimal recruitment of additional lung units at higher levels of PEEP [125] or recruitment without simultaneous improvement in PaCO_2,_ suggesting that recruited units are not functional/participating in gas exchange [126]. Indeed, higher levels of PEEP in C-ARDS have been reported to have deleterious effects on both gas exchange [121,127] and respiratory mechanics [121,125,127,128,129], consistent with net overdistension. In the advanced stages of C-ARDS when consolidation is extensive, even PEEP levels as low as 5 cmH_2_O may be associated with markedly elevated airway plateau and driving pressures [101].

These data serve to underscore the importance of tailoring PEEP to the patient’s individual physiology. To minimize the hemodynamic and mechanical risks associated with PEEP, it should only be increased if doing so leads to demonstrable recruitment of functional lung units. While all methods of PEEP titration are imperfect, targeting optimal compliance is a reasonable strategy. If an increase in PEEP results in improved system compliance (while tidal volume is held constant), aeratable lung capacity has increased and recruitment has occurred. Recruitment of functional lung units is additionally associated with reduced PaCO_2_ for a given minute ventilation as a result of decreased dead space ventilation and increased surface area for gas exchange; while physiologic dead space is not routinely measured in clinical settings, the ventilatory ratio correlates reasonably well [122], is easily measured, and can be tracked following adjustments in PEEP. Similarly, the recruitment to inflation (R/I) ratio is a bedside test that has been used to estimate lung recruitability in response to changes in PEEP [130].

### 8.3. Body Positioning

Lung tissue mass is not distributed evenly, with 60% being located in the dependent (dorsal) half of the sterno-vertebral axis when supine [131]. In ARDS, the dorsal lung is predisposed to compressive atelectasis when supine due to the weight of overlying edematous tissue. External compression of lower lung units by the abdominal contents and of medial lung units by the weight of the overlying heart may also occur [132,133]. Atelectasis results in relatively well-perfused but reversibly non-ventilated alveoli [134]. The ventral lung, on the other hand, is predisposed to overdistension during passive ventilation, not only because it receives a greater proportion of that ventilation, but also due to the increased regional compliance of the anterior chest wall (relative to the posterior chest wall), which permits a greater degree of end-tidal distension of adjacent lung units [135].

**Figure 2:**
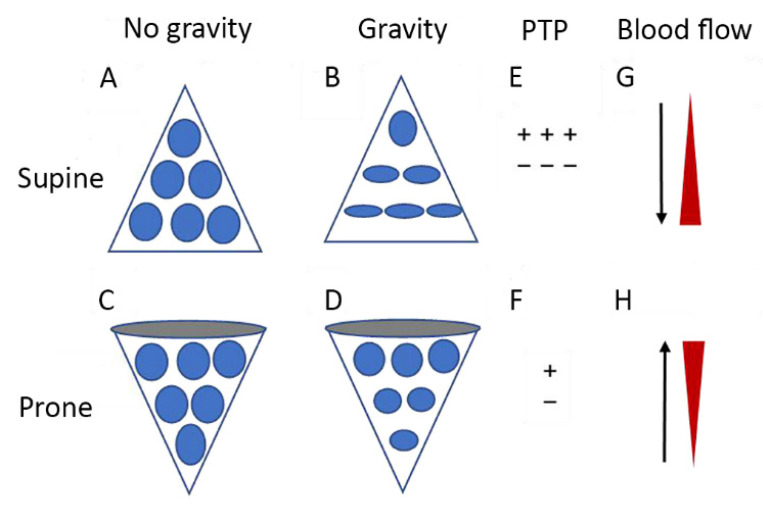
Diagrammatic presentation of physiological mechanisms associated with pronation in acute respiratory distress syndrome (ARDS). (**A**,**C**) show the shape of lung units (i.e., alveoli) without the effect of gravity. (**B**) In the supine position, the volume of dorsal lung units is significantly smaller than the volume of ventral lung units, as a result of gravity and pleural pressure; thus, ventral lung units are more prone to overdistention and dorsal lung units are more prone to compression atelectasis. (**D**) In the prone position, gravity and pleural pressure result in a decrease in the volume of the ventral lung units and an increase in the volume of the dorsal lung units. (**E**) In the supine position, the ventral transpulmonary pressure (PTP) may substantially exceed the dorsal PTP (**F**) Prone positioning reduces the ventral-to-dorsal PTP gradient thereby augmenting the homogeneity of ventilation. (**G**) The reopening, dorsal lung units continue to receive most of the blood flow. (**H**) The ventral lung units may exhibit a greater tendency to collapse, but are still relatively underperfused. Reproduced in concordance with the Creative Commons Attribution License (CC-BY) from [138].

In the prone position, previously compressed dorsal and medial lung units are recruited, and previously gas-filled ventral lung units become less distended or collapse altogether. Despite this tendency for collapse of ventral lung units, there is typically net recruitment, as the loss of ventral lung units is outweighed by recruitment of units in the dorsal region, which contains a greater mass of lung tissue [136]. Prone positioning further results in better anatomical matching of the lung and chest wall shapes and compliance along the vertical axis, leading to less variation in size of individual pulmonary units [135] (Figure 2). Since the distribution of lung perfusion remains virtually unchanged in the prone position, these changes result in more homogenous ventilation, with decreases in both venous admixture and dead space. Proning may also result in reduced lung stress (i.e., transpulmonary pressure) and strain (i.e., the tidal volume-to-end-expiratory lung volume ratio) [137], decreasing the risk of VILI.

The use of prone positioning has increased significantly during the COVID pandemic, with 77% of mechanically ventilated C-ARDS patients with a P/F < 100 being placed in the prone position [139] compared to only 16% of ARDS patients with a P/F < 100 during the pre-COVID era [140]. It remains one of the few interventions in severe ARDS associated with survival benefit, as demonstrated by a landmark study showing significant mortality reduction when patients with ARDS and a P/F < 150 were placed the prone position for least 16 h daily [141]. While that trial preceded the advent of COVID, recent investigations performed in C-ARDS patients also suggest a survival benefit, with one retrospective study demonstrating a small but statistically significant reduction in the risk of death when C-ARDS patients with a P/F < 200 were proned within the first 2 days of ICU admission [142].

Studies that have investigated the physiologic effects of prone positioning in C-ARDS patients have generally reported improved oxygenation, with P/F increasing ≥ 20 mmHg in approximately 75% of patients [139]. Responses to proning are heterogeneous though, and available data suggest that the mechanisms responsible for improved oxygenation may differ from those in usual ARDS.

Unlike typical ARDS, net recruitment of C-ARDS lungs following placement in the prone position is relatively modest and often negligible [143]. Improved system compliance, typically present when significant net recruitment occurs, has not been observed in most studies [139,143,144,145,146]. While measurements of partitioned respiratory mechanics would be needed to conclude with certainty that the lack of improvement in system compliance is not the result of decreased chest wall compliance in the prone position, counterbalancing a simultaneous increase in compliance of newly recruited lung, an absence of significant recruitment is suggested by other findings as well.

CO_2_ exchange often improves in the prone position as a result of decreased dead space and recruitment of additional lung units. Most studies that have evaluated gas exchange in the prone position in C-ARDS patients, however, have reported little change in the PaCO_2_ (or ventilatory ratio) [139,143,145,146]. Compared to typical ARDS, the changes in both respiratory system compliance and PaCO_2_ following prone positioning are significantly less in patients with C-ARDS [99]. In the absence of recruitment, the most plausible mechanism to explain improved oxygenation is better matching of ventilation/perfusion ratios of vaso-dysregulated tissue [136].

Timing may also play a significant role in response to prone positioning [99,143]. In unresolving ARDS, atelectasis and edema may evolve into significant dorsal consolidation and diffuse fibrosis; in this setting, there is minimal recruitment of dorsal tissue in the prone position—only increased ventral atelectasis. A significant percentage of such patients either experience worsened P/F ratio in the prone position or fail to meet the accepted criteria for “responsiveness” (improvement in P/F ≥ 20 mmHg) [145].

## 9. Extracorporeal Life Support for C-ARDS

Extracorporeal life support (ECLS) refers to supplemental gas exchange via an external circuit. ECMO provides sufficient blood flow rates for either respiratory gas exchange support alone (venovenous (VV) ECMO) or complemented by circulatory support (venoarterial (VA) ECMO). Extracorporeal carbon dioxide removal (ECCO_2_R) requires lower blood flow rates than ECMO and yet efficiently clears carbon dioxide (CO_2_). However, unlike ECMO, ECCO_2_R does not effectively re-oxygenate the mixed venous blood. ECMO requires the placement of a central venous cannula, attached to a circuit that pumps blood under negative pressure and delivers it to an “oxygenator” or “membrane lung” (i.e., a gas exchange device). Oxygen passing through the device’s hollow and gas-permeable fibers then transfers across them into the diverted venous blood flow, while CO_2_ is removed by diffusing from blood into the unidirectional stream of “sweep” gas passing through the fiber lumens. The membrane-oxygenated blood is then pumped back into the patient via a second intravascular cannula inserted so that its tip is placed close to the right atrium, or via the return channel of a dual-lumen cannula. Both types of external circuit require anticoagulation. Compared to ECMO, ECCO_2_R can be achieved using smaller catheters, thereby reducing the risk of cannulation-related complications. Such cannulation is usually percutaneous, using a modified Seldinger technique with imaging guidance. As the application of ECLS is clinically demanding, current evidence suggests that it should be performed in selected centers with adequate experience [147].

VV-ECMO has proven effective in patients with hypoxemia refractory to optimized mechanical ventilation settings and adjunctive therapies, including prone positioning [148,149]. Current criteria for initiating ECMO in C-ARDS are those previously used in the ECMO to Rescue Lung Injury in Severe ARDS (EOLIA) trial, and comprise: a P/F of <80 mmHg for >6 h; a P/F of <50 mmHg for >3 h; or an arterial pH of <7.25 with PaCO_2_ ≥ 60 mmHg for >6 h [147,148]. In the EOLIA trial, patients with the abovementioned criteria were randomized either to receive VV-ECMO or to continue treatment with conventional mechanical ventilation [150]. Although the study did not show a statistically significant difference in 60-day mortality between the two groups, the effect estimate favored the intervention group (relative risk, 0.76; 95% confidence interval, 0.55–1.04; P = 0.09). In addition, 28% of patients of the control group were crossed over to VV-ECMO for refractory hypoxemia. These facts could still imply a likely VV-ECMO mortality benefit. Notably, enrolled patients had to be on mechanical ventilation for < 7 days while other adjunctive therapies, including inhaled nitric oxide, recruitment maneuvers, high-frequency oscillatory ventilation, and infusion of almitrine were also allowed [150]. While the majority of the patients had ARDS caused by pneumonia, bacterial (45%) or viral (18%), approximately 37% of the study population had ARDS of other etiology.

In accordance with current guidelines, major contraindications to VV-ECMO could include prolonged (i.e., >10 days) mechanical ventilation, morbid obesity or advanced age (e.g., >75 years), chronic respiratory failure, heart failure requiring VA-ECMO, heparin-induced thrombocytopenia, cancer with life expectancy of <5 years, a moribund condition or a Simplified Acute Physiology Score II of >90, non-drug-induced coma after cardiac arrest, irreversible neurologic injury, the presence of a treatment limitation decision, patient/surrogate decline of blood products, expected difficulty with vascular cannulation, and unavailability of adequate specialized staff and ECMO equipment [150,151]. Current recommendations do not advise any deviations from conventional ECMO practices applied to ARDS patients without COVID-19, including anticoagulation [151].

The initial goal of ECMO is to maintain adequate oxygenation. However, the primary mechanism of benefit may be a decreased risk of VILI, the result of membrane CO_2_ clearance, lower driving and plateau pressures, and reduced tidal volumes and inflation power used during ECMO [149]. Additional clinical goals of ECMO might include minimization of sedation, early weaning from mechanical ventilation, and patient mobilization. However, in C-ARDS, discontinuation of sedation could be potentially followed by extreme patient respiratory effort and risk P-SILI [152] There currently is insufficient evidence to support or refute early mobilization or extubation with awake ECMO in this setting [151].

Except for very severe ARDS, where hypoxemia is the major concern, there is a rationale for lowering ventilation volumes and pressures beyond standard values to reduce further the risk of VILI. As doing so may lead to hypercapnic acidosis, ECCO_2_R can be used to avoid impermissible hypercapnia [153]. In April 2020, emergency authorization was issued for the use of an ECCO_2_R device in patients with C-ARDS, with or without mechanical ventilation [154]. This approach is not only physiologically sound, but also can be less costly and technically easier to apply than ECMO. Nevertheless, in a multicenter RCT of typical ARDS, the use of ECCO_2_R was not associated with a reduction in 90-day mortality [155].

The effect of ECMO on outcomes of C-ARDS patients has been evolving and repeatedly evaluated during the pandemic. In a 2021 systematic review of 22 observational studies, ECMO outcomes of C-ARDS were similar to those of non-COVID-19 patients [156]. However, subsequent studies and meta-analyses reported rises of up to 15% in the mortality rates during the second wave as well as after the first year of the pandemic [157,158,159]. This finding has been attributed to (1) the evolving viral strains of SARS-CoV-2; (2) the evolution of pharmacological treatment during the later phases of the pandemic (e.g., the addition of immunomodulatory therapies); and (3) the broader and longer use of non-invasive ventilation in the C-ARDS population [158]. Changes in COVID-19 treatment strategies may have contributed to the selection of patients with refractory disease and/or more severe P-SILI for ECMO treatment [157]. The main risk factors for mortality reported in the literature for C-ARDS patients on ECMO include older age, the presence of multiple comorbidities and systemic acidosis, the need for renal replacement therapy and high vasopressor infusion rates, and finally the occurrence of bleeding complications [156,160]. Other frequent complications include thromboembolic events, infections, ventilator-associated pneumonia, bacteremia, and ECMO circuit-related mechanical problems [157,158]. The occurrence of neurological complications and in particular of intracranial hemorrhage, although rare, affecting 6–12% of the patients, has been associated with a mortality rate of 90%, implying that C-ARDS patients on ECMO could benefit from early non-invasive neuromonitoring protocols [161].

Despite the similar guidelines for patient selection and implementation of ECMO in C-ARDS and typical ARDS, there are some significant differences between these two groups. There are more practical difficulties affecting the medical and nursing teams caused by the risk of transmission and the use of personal protective equipment during C-ARDS ECMO. C-ARDS patients have a higher risk of thrombosis and a higher prevalence of right ventricular failure [162]. The use of immunomodulatory and/or immunosuppressive therapies is also more frequent in C-ARDS, and a *longer* duration of support is likely to be necessary for these patients [163]. It is currently acknowledged that successful lung recovery is possible after prolonged VV-ECMO (>28 days) [164]. Distinct clinical courses of C-ARDS ECMO have been described. Some patients with C-ARDS achieve lung recovery shortly after the initiation of ECMO (i.e., within a few days), while others require prolonged ECMO, posing unique clinical challenges. While a delayed lung recovery may be possible in such patients on prolonged ECMO, alternative trajectories such as lung transplantation, or transition to comfort-based care, may need to be considered [151,165].

The combination of prone positioning and ECMO (PP-ECMO) may also be considered [166,167]. In a physiological study of C-ARDS, PP-ECMO was associated with higher P/F ratio, better respiratory system compliance, and lower driving pressure and mechanical power of ventilation; improvements in respiratory mechanics persisted after supine repositioning [168]. In a retrospective, multicenter, cohort study of C-ARDS patients featuring propensity score matching, PP-ECMO was associated with improved oxygenation, reduced intrapulmonary shunt, tolerance of longer ECMO duration, and lower in-hospital mortality [169]. Further high-quality research is still needed to determine the potential clinical usefulness of PP-ECMO in C-ARDS. Table 3 summarizes currently available therapeutic options for C-ARDS.

## 10. Conclusions

Typical ARDS is characterized by high-permeability edema, widespread atelectasis, and a loss of compliance that relates directly to the reduced capacity of aerated lung units. COVID-19, a novel etiology of ARDS, has distinct pathologic findings consistent with severe injury to—and dysfunction of—the pulmonary vasculature as a result of SARS-CoV-2-induced endothelial injury and immunothrombosis. The lungs of patients with C-ARDS may be more likely to overdistend than to recruit in response to customary levels of PEEP. A subpopulation of patients with C-ARDS may present with severely deranged gas exchange that is uncoupled from the comparatively mild parenchymal injury. Just as typical ARDS encompasses a broad range of clinical findings, so too does C-ARDS, often transitioning in its more advanced stages to a form indistinguishable from typical ARDS. Some have argued that all patients with ARDS, regardless of etiology, should be treated identically. This approach, however, ignores the physiologic variability that not only exists between patients, but also within individual patients depending on the phase of the disease.

Randomized trials in ARDS have identified several interventions that lead to improved outcomes. These studies have enrolled patients with significant heterogeneity though and as such, a significant degree of heterogeneity in treatment effect is to be expected [170]. They report the mean intervention effects observed in a population, but with regard to benefit, wide individual variability exists. Randomized trials have provided safe starting points from which to approach mechanical ventilation in the individual, but such rules are not inviolable. A more holistic approach, taking into consideration the unique physiology of individual patients, is warranted—as exemplified by C-ARDS.

## Figures and Tables

**Table 1:** Berlin Definition of Acute Respiratory Distress Syndrome. *CXR*, chest X-ray; *CT*, computed tomography; *PaO_2_/FiO_2_*, partial pressure of arterial oxygen to fraction of inspired oxygen ratio; *PEEP*, positive end-expiratory pressure; *CPAP*, continuous positive airway pressure.

**Timing**		Within 1 week of known clinical insult or new or worsening respiratory symptoms
**Chest imaging**		Bilateral opacities on CXR or CT not fully explained by effusions, lobar/lung collapse, or nodules
**Origin of edema**		Respiratory failure not fully explained by cardiac failure or fluid overload
**Oxygenation**	*Mild*	200 mm Hg < PaO_2_/FiO_2_ ≤ 300 mm Hg with PEEP or CPAP ≥ 5 cm H_2_O
	*Moderate*	100 mm Hg < PaO_2_/FiO_2_ ≤ 200 mm Hg with PEEP ≤ 5 cm H_2_O
	*Severe*	PaO_2_/FiO_2_ ≤ 100 mm Hg with PEEP ≥ 5 cm H_2_O

**Table 2:** Comparative presentation of major characteristic features of typical ARDS and C-ARDS. *ARDS*, acute respiratory distress syndrome; *C-ARDS*, coronavirus disease (COVID) 19-related ARDS; *SARS-CoV-2*, severe acute, respiratory syndrome coronavirus 2; *PaO_2_/FiO_2_*, oxygen arterial partial pressure-to-fraction of inspired oxygen fraction ratio; *PEEP*, positive end-expiratory pressure; *ECMO*, extracorporeal membrane oxygenation. * May predispose to early, profound hypoxemia and the conceptual risk of pre-intubation, patient self-inflicted lung injury.

	Typical ARDS	C-ARDS
Etiology	Diverse, pulmonary or extrapulmonary (e.g., bacterial or viral pneumonia, severe trauma, aspiration, sepsis, etc.)	SARS-CoV-2 infection of alveolar type 2 cells (primarily)
Hypoxemia (PaO_2_/FiO_2_ ≤ 300 mmHg at a PEEP level of ≥ 5 cmH_2_O)	Acute onset (e.g., within <48 h after the clinical insult), or progressive onset (i.e., within 7 days after the clinical insult)	Progressive onset (i.e., within 7 or more days after the onset of COVID-19 symptoms) *
Lung compliance at hypoxemia onset	Usually low (e.g., <40 cmH_2_O/L)	Usually high (e.g., >40 cmH_2_O/L)
Recruitment potential	Low or high, depending on the extent/nature of lung unit involvement and associated atelectasis	Initially low—may increase with disease progression and development of edema and atelectasis
Functional-to-anatomical shunt ratio/hyperperfusion of gasless tissue *	Usually 0.5–2.0/no	Usually > 2.0/yes
Alveolar capillary microthrombosis/new vessel growth	Present/present	Diffuse (~9 times more prevalent)/marked (2.7 times higher)
Clinical benefit from lung-protective ventilation	Proven	Highly likely
Clinical benefit from prone positioning	Proven	Highly likely
Clinical benefit from corticosteroids	Likely; more high-quality evidence needed	Proven
Clinical benefit from targeted anti-inflammatory interventions	Uncertain; lack of intervention-specific evidence	Proven
Clinical benefit from ECMO	Likely	Possible; high-quality evidence still needed

**Table 3:** Evidence-based treatments for coronavirus disease 19 (COVID-19)-related acute respiratory distress syndrome (ARDS). *PaO_2_/FiO_2_*, oxygen arterial partial pressure-to-inspired oxygen fraction ratio; *PEEP*, positive end-expiratory pressure. * Time interval corresponds to the maximum recommended duration of therapy. ^†^ To be reduced to 2 mg if estimated glomerular filtration rate is 60 mL/min or less.

Intervention	Mechanism of Action	Evidence for Efficacy
Remdesivir day 1: 200 mg IV days 2–10: 100 mg IV	Inhibition of the viral RNA-dependent, RNA polymerase	Shortens the time to recovery in hospitalized COVID-19 patients
Dexamethasone days 1–10 *: 6 mg IV	Anti-inflammator linked to the activation of the glucocorticoid receptor	Reduces the probability of in-hospital death in critically ill COVID-19 patients
Tocilizumab single dose: 8 mg/kg IV (max. 800 mg)	Interleukin 6 antagonism	Reduces the probability of in-hospital death in critically ill COVID-19 patients
Baracitinib days 1–14 *: 4 mg ^†^ oral or enteral	Janus kinase inhibition	Reduces the probability of in-hospital death in critically ill COVID-19 patients
Anakinra days 1–10 *: 100 mg subcutaneously	Interleukin 1 alpha/beta antagonism	Reduces the probability of in-hospital death in critically ill COVID-19 patients
Prone positioning for at least 16 h per day until PaO_2_/FiO_2_ ≥150 mmHg at PEEP ≤10 cmH_2_O and FiO_2_ ≤ 0.6	Attenuation of lung stress and strain Reversal of compression atelectasis Increased homogeneity of ventilation Improved ventilation/perfusion matching	Reduces the probability of in-hospital death in moderate to severe ARDS
Extracorporeal membrane oxygenation	Minimization of lung stress and strain (“lung rest”) with very low tidal volumes and ventilation pressures	Possible mortality benefit in severe ARDS
